# The geological record and phylogeny of spider wasps (Hymenoptera: Pompilidae): A revision of fossil species and their phylogenetic placement

**DOI:** 10.1371/journal.pone.0185379

**Published:** 2017-10-11

**Authors:** Juanita Rodriguez, Cecilia Waichert, Carol D. von Dohlen, James P. Pitts

**Affiliations:** 1 CSIRO, Australian National Insect Collection, Acton, ACT, Australia; 2 Departamento de Ciências Biológicas, Programa de Pós-Graduação em Ciências Biológicas, Universidade Federal do Espírito Santo, Vitória ES, Brazil; 3 Utah State University, Department of Biology, Logan, UT, United States of America; Institute of Botany, CHINA

## Abstract

Accurate fossil identification has become increasingly relevant with the widespread use of phylogenetic divergence time estimation methods, which rely on fossil data to determine clade hard-minimum ages. Here we revise, diagnose and illustrate known spider wasp (Hymenoptera: Pompilidae) fossil species and place them within the latest Pompilidae phylogenetic hypothesis. *Ceropalites infelix* Cockerell, from the Florissant Fossil Beds (Priabonian), is no longer recognized as Pompilidae, but as Aulacidae. *Agenioideus saxigenus* (Cockerell) **comb. nov.**, *Deuteragenia wettweri* (Statz) **comb. nov.**, *Caputelus scudderi* (Cockerell, 1906) **comb. nov.**, *Pepsinites avitula* (Cockerell, 1941) **comb. nov.**, *Pepsinites contentus* (Theobald, 1937) **comb. nov.**, *Pepsinites florissantensis* (Cockerell, 1906) **comb. nov.**, *Pepsinites laminarum* (Rohwer, 1909) **comb. nov.**, *Pepsinites scelerosus* (Meunier, 1919) **comb. nov.**, *Pepsinites cockerellae* (Rohwer, 1909) **comb. nov.**, *Pompilinites coquandi* (Theobald, 1937) **comb. nov.**, *Pompilinites depressus* (Statz, 1936) **comb. nov.**, *Pompilites incertus* (Theobald, 1937) **comb. nov.,** *Pompilites induratus* (Heer, 1849) **comb. nov.,** *Pompilites fasciatus* (Theobald, 1937) **comb. nov.,** and *Pompilites senex* **comb. nov.** are new combinations. Twenty-three fossil species of spider wasps are now recognized in 13 genera. Four new genera are proposed: *Caputelus* Waichert & Pitts **gen. nov.,** *Pompilites* Rodriguez **gen. nov.,** *Pompilinites* Rodriguez & Waichert **gen. nov.**, and *Pepsinites* Rodriguez & Waichert **gen. nov.**, of which the three latter are collective-group names for fossils with taxonomic uncertainty. One species of fossil spider wasp is described: *Deuteragenia catalunyia* Rodriguez, Waichert & Pitts **sp. nov.**, from the Bellver deposits in Catalonia, Spain. Five of the 23 known species can be used to determine hard-minimum age for calibrations of genera stem-groups (*Agenioideus*, *Anoplius*, *Cryptocheilus*, *Deuteragenia*, *Priocnemis*). The fossil belonging to the stem-group of the tribe Ageniellini (*Chubutholites*) is not recommended for calibration because of the high uncertainty in its age and taxonomy. The remaining taxa can be assigned to the lineage comprising Pompilinae + Pepsinae (12 species) or crown-group Pompilidae (four species).

## Introduction

Fossil species identification is becoming more relevant with the widespread use of molecular data for phylogenetics and the possibility of producing time-calibrated trees. Unfortunately, many fossilized specimens lack relevant preserved structures necessary for classification at the genus and species level, which makes taxonomically difficult groups even more challenging. Spider wasps (Hymenoptera: Pompilidae), for instance, have a puzzling taxonomy, because of morphological uniformity. Pompilid fossil taxa have an additional issue and it is the lack of appropriate preserved structures, and descriptions which mostly date from the early 1900s, where the majority of images accompanying species descriptions are poor or non-existent.

Fossil spider wasps have not been revised as a whole. The most recent work described Dominican amber species and excluded a Cretaceous fossil species from the family [1]. This paleontological study corroborated molecular phylogenetic analyses, showing that the oldest Pompilidae are from the Eocene and not the Cretaceous, as was previously proposed [1,2]

Here we provide a revision of the existing spider wasp fossils and place them as potential calibration points on the most recent phylogeny of the group [2].

## Methods

### Taxonomic revision

Compression and amber fossils from various natural history collections were studied. The compression fossils studied belong to seven main deposits: the Florissant Fossil Beds (Florissant, Colorado, USA), the Oeningen deposits (Baden-Württemberg, Germany), the Rott deposits (Rhineland-Palatinate, Germany), the Aix-en-Provence deposits (Bouches-du-Rhône, France), the Terrains sannoisiens du Gard deposits (Gard, France), Camoins-les-Bains (Marseille, France) and the Bellver deposits (Lleyda, Spain). The Dominican amber fossils studied derive from deposits found in mines between the cities of Santiago and Puerto Plata (Dominican Republic). One of the Baltic amber fossils derives from the Kaliningrad region (Russia). The locality of the second Baltic amber fossil is unknown.

Abbreviations used are the same as those by Wasbauer & Kimsey [3]. They are defined as follows: LA3, length of third antennal segment and WA3, width of third antennal segment. Measurements of the clypeus are as follow: WC, width of clypeus, measured between the widest points; and LC, maximum height of clypeus. Wing venation terminology follows that of Huber & Sharkey [4]. Pictures were either provided by curators or taken with a Jenoptik camera coupled to a Leica Mz7.5 microscope; line drawings were processed in Adobe Illustrator. The kind of material studied is summarized in Table 1.

**Table 1:** Species of fossil Pompilidae, material type, type location and age.

Subfamily		Species name after this revision	Material	Type	Occurrence	Age
Pompilinae	1	*Agenioideus saxigenus* (Cockerell, 1908) **comb. nov.**	Holotype	Compression	Florissant Fossil Beds	Priabonian
	2	*Anoplius planeta* Rodriguez & Pitts, 2016	Holotype	Amber	Dominican amber	Burdigalian to Langhian
	3	*Tainopompilus argentum* Rodriguez & Pitts, 2016	Holotype	Amber	Dominican amber	Burdigalian to Langhian
	4	*Tenthredinites bifasciata* Meunier, 1915	Literature	Compression	Aix-en-Provence	Chattian
Pompilinae	5	*Pompilinites coquandi* (Theobald, 1937) **comb. nov.**	Holotype	Compression	Aix-en-Provence	Chattian
*incertae sedis*	6	*Pompilinites depressus* (Statz, 1936) **comb. nov.**	Literature	Compression	Rott deposits	Chattian
Pepsinae	7	*Chubutolithes gaimanensis* Bown & Ratcliffe, 1988	Literature	Ichnofossil	Sarmiento Formation	Priabonian
	8	*Cryptocheilus hypogaeus* Cockerell, 1912	Holotype	Compression	Florissant Fossil Beds	Priabonian
	9	*Deuteragenia catalunyia* Rodriguez, Waichert & Pitts **sp. nov.**	Holotype	Compression	Bellver deposits	Messinian
	10	*Deuteragenia wettweri* (Statz, 1938) **comb. nov.**	Holotype	Compression	Rott deposits	Chattian
	11	*Paleogenia wahisi* Waichert & Pitts, 2016	Holotype	Amber	Baltic amber	Priabonian
	12	*Priocnemis aertsi* Statz, 1936	Holotype	Compression	Rott deposits	Chattian
	13	*Caputelus scudderi* (Cockerell, 1906) **comb. nov.**	Holotype	Compression	Florissant Fossil Beds	Priabonian
Pepsinae	14	*Pepsinites scelerosus* (Meunier, 1919) **comb. nov.**	Literature	Amber	Baltic amber	Lutetian to Priabonian
*incertae sedis*	15	*Pepsinites avitula* (Cockerell, 1941) **comb. nov.**	Holotype	Compression	Florissant Fossil Beds	Priabonian
	16	*Pepsinites florissantensis* (Cockerell, 1906) **comb. nov.**	Holotype	Compression	Florissant Fossil Beds	Priabonian
	17	*Pepsinites laminarum* (Rohwer, 1909) **comb. nov.**	Holotype	Compression	Florissant Fossil Beds	Priabonian
	18	*Pepsinites contentus* (Theobald, 1937) **comb. nov.**	Literature	Compression	Terrains sannoisiens du Gard	Priabonian
	19	*Pepsinites cockerellae* (Rohwer, 1909) **comb. nov.**	Holotype	Compression	Florissant Fossil Beds	Priabonian
Pompilidae	20	*Pompilites induratus* (Heer, 1849) **comb. nov.**	Holotype	Compression	Oeningen	Langhian to Serravallian
*incertae sedis*	21	*Pompilites fasciatus* (Theobald, 1937) **comb. nov.**	Holotype	Compression	Aix-en-Provence	Chattian
	22	*Pompilites incertus* (Theobald, 1937) **comb. nov.**	NL	Compression	Camoins-les-Bains	Chattian
	23	*Pompilites senex* (Rohwer, 1909) **comb. nov.**	Holotype	Compression	Florissant Fossil Beds	Priabonian

Ages according to the Geological Society of America timescale v. 4.0. (http://www.geosociety.org/documents/gsa/timescale/timescl.pdf)

The species treated here were assigned to the family Pompilidae based mainly on wing venation features, which are relatively uniform for the family [5]. All of the specimens studied have a preserved forewing, and most of them have the hindwing also preserved (Table 1). They were placed in the family Pompilidae based on a combination of wing venation character states [5–7]: forewing with ten closed cells, C vein present, 1Rs not directly joining pterostigma, abscissa distad Rs + M from M vein present, veins 2rs-m and 3rs-m present, 2m-cu present, 1cu-a closer to vein 1M than to its junction with Cu vein, 2-Rs vein present; hindwing with distinct claval lobe absent, first abscissa of Cu present, 1A vein absent, 1rs-m crossvein absent, second abscissa of M present, and the veins C+Sc+R+Rs fused basally. Pompilidae shares most of Sharkey & Roy’s [6] wing venation character states with Tiphiidae and Sapygidae, but Tiphiidae have a distinct claval lobe [7] and Sapygidae have a vein 1Rs that joins or is closer to the pterostigma [6]. Additionally, Tiphiidae usually have a cilindrical metasoma and males with spined hypopygium, and in both Tiphiidae and Sapygidae the hind leg femur does not surpass the metasoma.

The acronyms for the collections used in this study are as follows:

**Table:** 

AMNH	American Museum of Natural History, New York, New York, USA
LACMIP	Los Angeles County Museum of Invertebrate Paleontology, Los Angeles, California, USA
MCZC	Museum of Comparative Zoology, Harvard University, Cambridge, Massachusetts, USA
MGMM	Museo Geominero de Madrid, Madrid, Spain
MHNM	Muséum d'histoire naturelle de Marseille, Marseilles, France
MHNN	Muséum d'Histoire naturelle–Nîmes, Nimes, France
MNHN	Muséum National d'Histoire Naturelle, Paris, France
OSAC	Poinar Amber Collection, Oregon State University, Corvallis, Oregon, USA
SMNK	Staatliches Museum für Naturkunde Karlsruhe, Karlsruhe, Germany
UCMC	University of Colorado Museum of Natural History, Boulder, Colorado, USA
USNM	Smithsonian National Museum of Natural History, Washington, District of Columbia, USA.

### Nomenclatural acts

The electronic edition of this article conforms to the requirements of the amended International Code of Zoological Nomenclature, and hence the new names contained herein are available under that Code from the electronic edition of this article. This published work and the nomenclatural acts it contains have been registered in ZooBank, the online registration system for the ICZN. The ZooBank LSIDs (Life Science Identifiers) can be resolved and the associated information viewed through any standard web browser by appending the LSID to the prefix “http://zoobank.org/”. The LSID for this publication is: urn:lsid:zoobank.org:pub: 260A7C4F-2B6E-44FC-87E0-033297134EF4. The electronic edition of this work was published in a journal with an ISSN, and has been archived and is available from the following digital repositories: PubMed Central and LOCKSS.

### Phylogenetic placement

After identification, fossils were placed onto the most recent molecular phylogeny [2] by morphologically matching the lowest reliable Linnaean taxonomic category with a monophyletic group and locating it on the stem group. The topology aims to provide and clarify the current data on pompilid fossils and phylogenetic relationships for future studies that might require chronological analyses. A conservative placement was used because none of the species can be matched to extant species, and therefore using them as crown-group calibrations could lead to error. It has been demonstrated that applying fossils to crown groups can inflate inferred ages [8]. We also provided in the topology hard minimum age values (lowest age of oldest fossil) to be used as priors in divergence time estimation analyses.

## Results and discussion

### Phylogenetic placement

**Fig 1:**
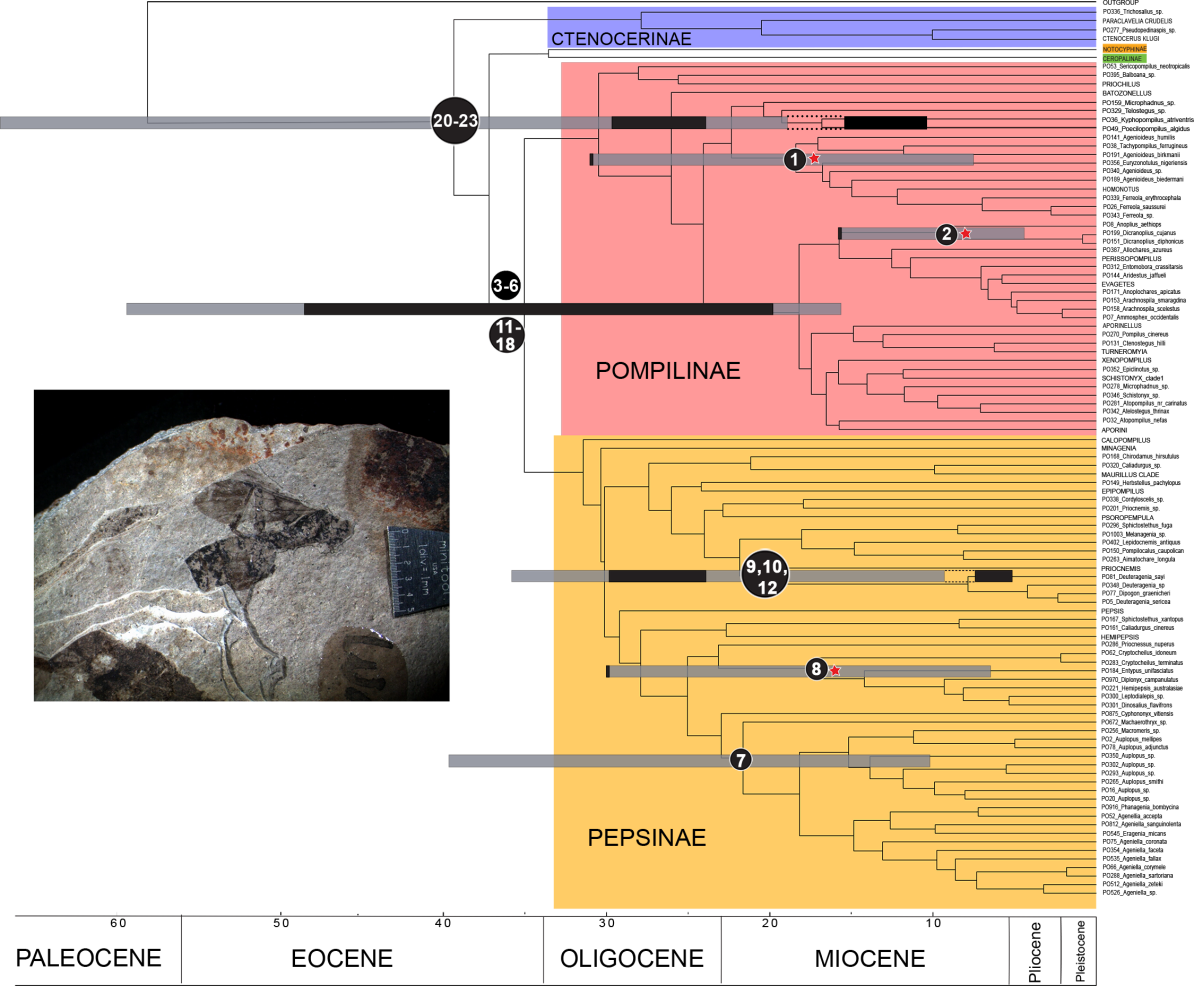
Molecular chronogram of Pompilidae (modified from Waichert *et al*. 2015) with geologic epochs indicated on the scale axis. Terminals in uppercase represent collapsed clades. Grey color represents 95% Highest Posterior Density (95%HPD) for node ages produced by the BEAST analysis in Waichert *et al*. (2015). Numbers on nodes correspond to fossil numbers in Table 1. Black shaded areas on node bars represent fossil age intervals. Nodes with stars were calibrated by Waichert *et al*. (2015). Dotted lines connect node bars to fossils falling outside the 95%HPD. Geological epochs on scale axis according to the Geological Society of America timescale v. 4.0. (http://www.geosociety.org/documents/gsa/timescale/timescl.pdf).

We revised 23 species, of which 18 are compression fossils, one is an ichnofossil (i.e. geological record of biological activity consisting on impressions or fossilized organic material) and four are preserved in amber (Table 1). Age information from these fossils was used to determine hard minimum age for future calibration studies. We plotted these bounds on the topology from Waichert *et al*. [2]. All fossil date ranges fell within estimated age error bars (Fig 1). Five fossils were useful in calibrating nodes at the genus stem-group level: *Agenioideus saxigenus* (Cockerell, 1908), *Anoplius planeta* Rodriguez & Pitts, 2016, *Cryptocheilus hypogaeus* Cockerell, 1912, *Priocnemis aertsii* Statz, 1936, and *Deuteragenia wettweri* (Statz, 1938) **comb. nov.** The first three were used by Waichert *et al*. [2] as calibration points in their analyses. Even though *Deuteragenia catalunyia* **sp. nov.** is confidently placed at the genus level, its age is younger than *D*. *wettweri* and, as such, it was not used to determine hard minimum age of *Deuteragenia* stem-group. Based on stratigraphic age uncertainty, the suggested hard-minimum ages for these genera are: 34.7 Ma for *Agenioideus* stem-group, 15 Ma for *Anoplius* stem-group, 34.7 for *Cryptocheilus* stem-group, and 28 Ma for *Priocnemis* and *Deuteragenia* stem-groups. A single fossil species–*Chubutolithes gaimanensis* Bown & Ratcliffe, 1988– can be placed as a calibration for stem-group Ageniellini. Because of uncertainty in its classification and age, we do not recommend its use to determine the hard-minimum age for this node (see *Chubutolithes gaimanensis* remarks section). Two species could be assigned to Pepsinae stem-group and eight to Pompilinae stem-group. Because of the sister relationship between Pepsinae and Pompilinae, these fossils were used to determine the hard-minimum age for their common ancestor. The oldest date from these fossils, taking into account age uncertainty, is 47.8 Ma based on the oldest possible age for *Paleogenia wahisi* Waichert & Pitts and *Pepsinites scelerosus* Meunier, 1919, from Baltic amber. Finally, the four Pompilidae *incertae sedis* are placed as crown-group Pompilidae in the absence of enough evidence to place them elsewhere. These suggest a hard-minimum age for Pompilidae of 28 Ma, based on the oldest two fossils: *Pompilites fasciatus* (Theobald, 1937) **comb. nov.** and *Pompilites incertus* (Theobald, 1937) **comb. nov.** This age, however, is younger than the suggested hard-minimum age of the most recent common ancestor of Pompilinae and Pepsinae, and therefore is not recommended as a calibration point.

### Systematic paleontology

Family **Pompilidae** Latreille, 1804

Subfamily **Pompilinae** Latreille, 1804

Genus ***Agenioideus*** Ashmead, 1902

**Fig 2:**
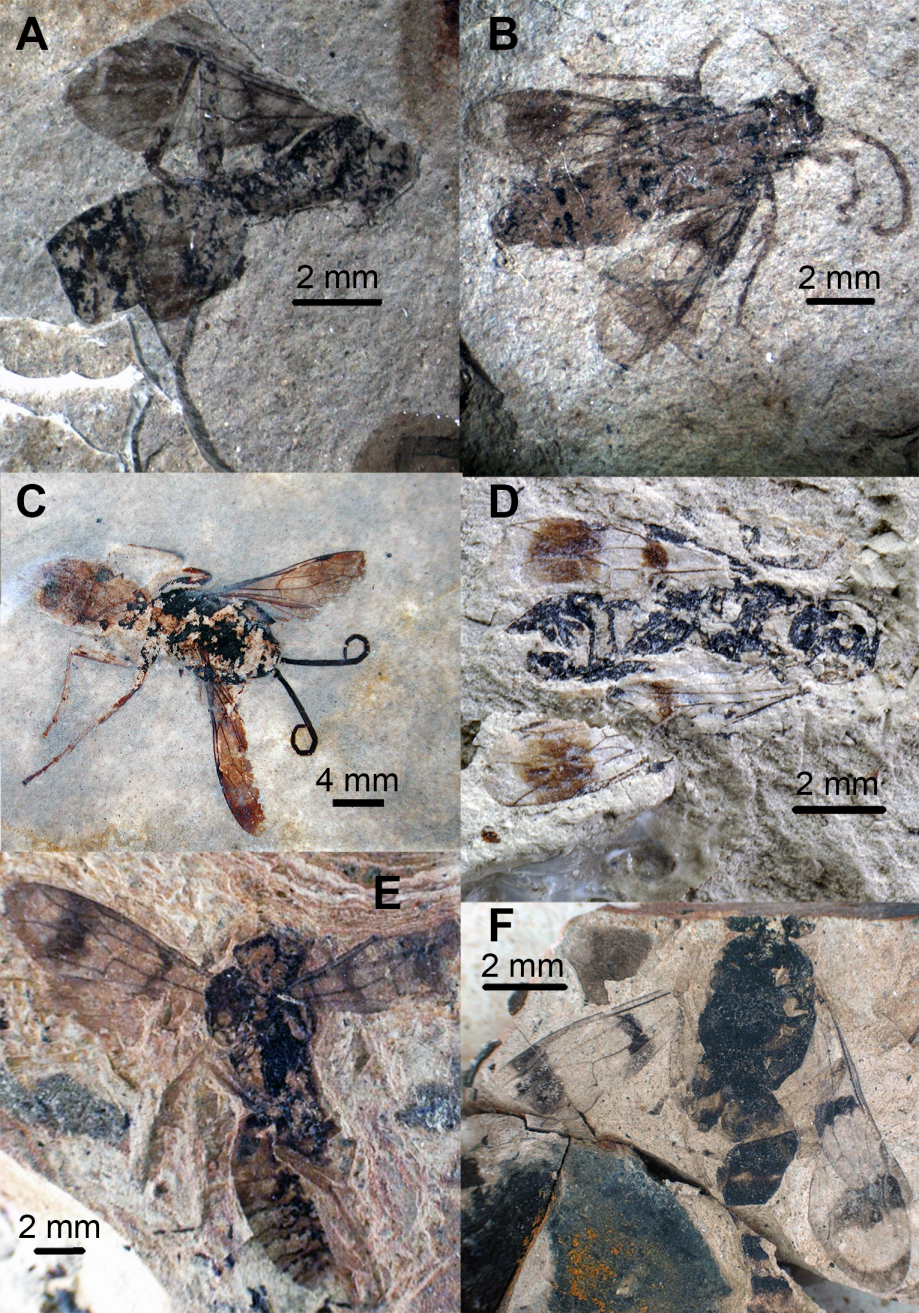
Photographs of *Agenioideus saxigenus*, *Pompilinites coquandi*, *Deuteragenia catalunyia*, *Deuteragenia wettweri* and *Priocnemis aertsi* type specimens. A, B, *Agenioideus saxigenus* (Cockerell, 1908) from Florissant Fossil Beds, Colorado, US (UCMC No. 4541): A, Holotype habitus, B, Paralectotype dorsal view; C, dorsal view of *Pompilinites coquandi* Theobald, 1937 from Aix-en-Provence deposits (MNHN); D, dorsal view *of Deuteragenia catalunyia* Rodriguez, Waichert & Pitts n. sp. from lacustrine deposits of Bellver, Spain (MGMM No. 2927M); E, habitus of *Deuteragenia wettweri* (Statz, 1938) from Rott deposits, Germany (LACMIP No. 3973); F, dorsal view of *Priocnemis aertsi* Statz from Rott deposits, Germany (LACMIP No. 3972).

**Fig 3:**
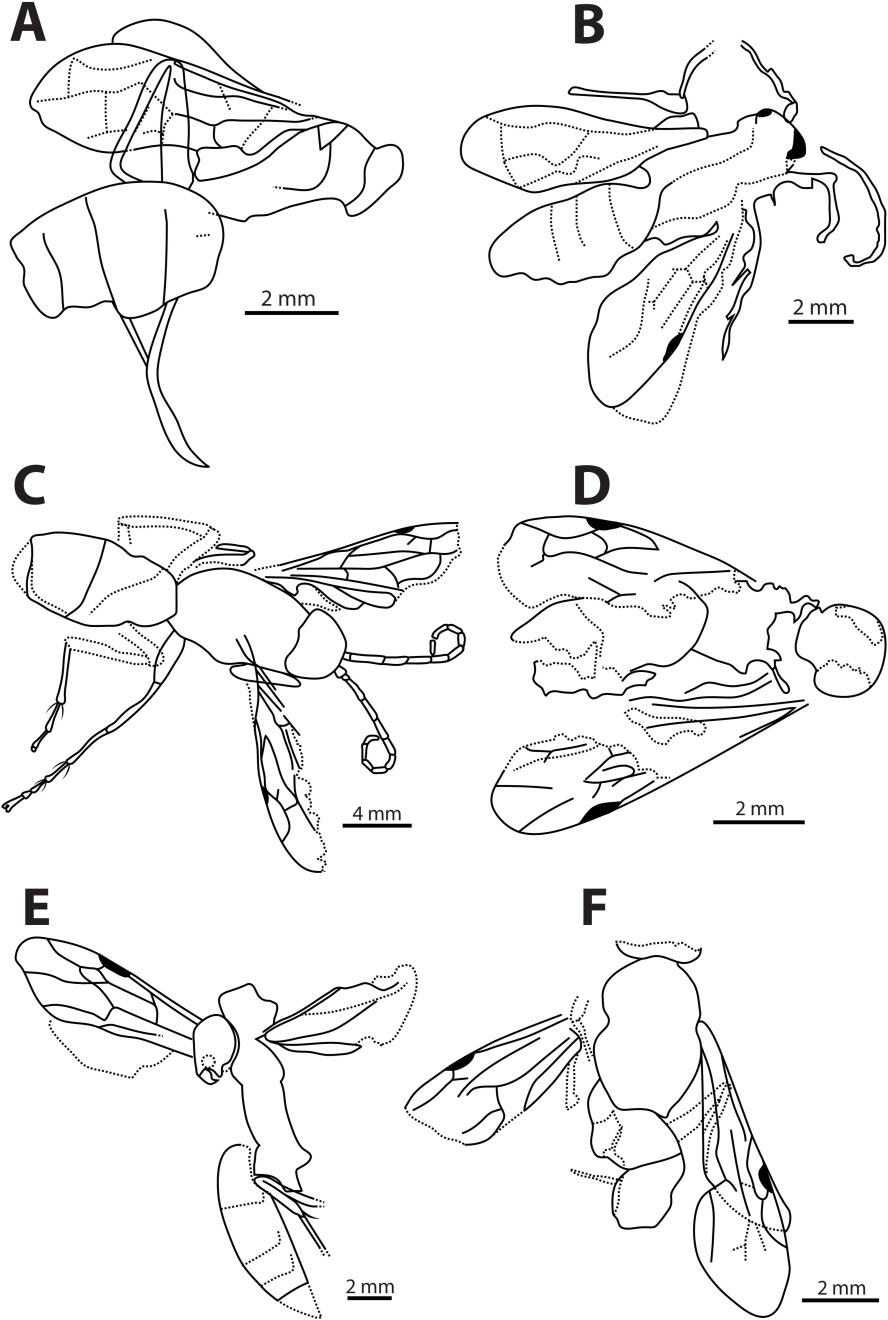
Line drawings of *Agenioideus saxigenus*, *Pompilinites coquandi*, *Deuteragenia catalunyia*, *Deuteragenia wettweri* and *Priocnemis aertsi* type specimens. A, B, *Agenioideus saxigenus* (Cockerell, 1908) from Florissant Fossil Beds, Colorado, US (UCMC No. 4541): A, Holotype habitus, B, Paralectotype dorsal view; C, dorsal view of *Pompilinites coquandi* Theobald, 1937 from Aix-en-Provence deposits (MNHN); D, dorsal view *of Deuteragenia catalunyia* Rodriguez, Waichert & Pitts n. sp. from lacustrine deposits of Bellver, Spain (MGMM No. 2927M); E, habitus of *Deuteragenia wettweri* (Statz, 1938) from Rott deposits, Germany (LACMIP No. 3973); F, dorsal view of *Priocnemis aertsi* Statz from Rott deposits, Germany (LACMIP No. 3972).

**Fig 4:**
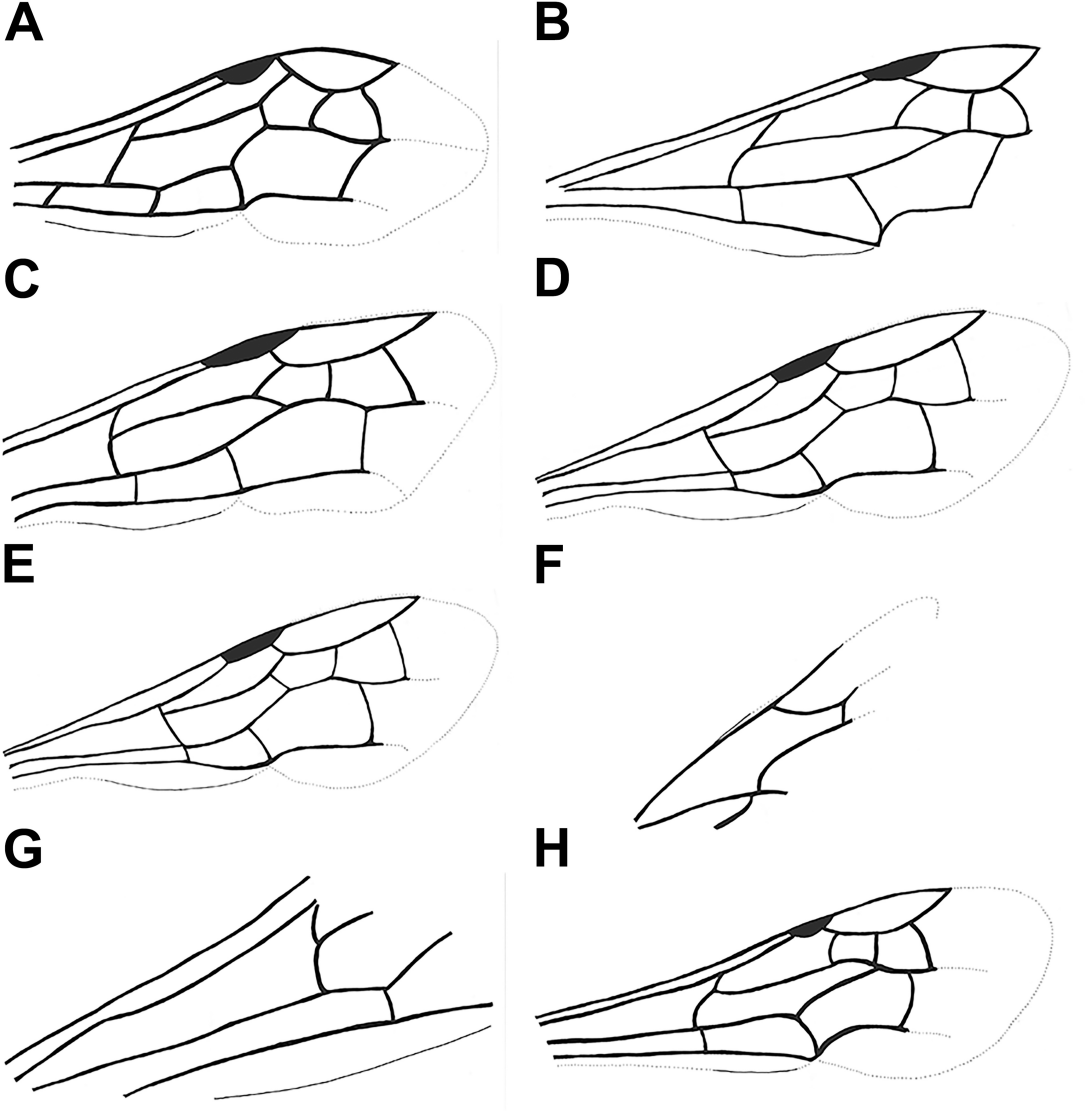
Wing drawings. A, forewing *Agenieoideus saxigenus*; B, forewing *Pompilinites coquandi*; C, forewing *Deuteragenia wettweri*; D, forewing *Priocnemis aertsi*; E, F, *Cryptocheilus hypogaeus*: E, forewing, F, hindwing; G, forewing *Pepsinites avitula*; H, forewing *Pompilites fasciatus*.

***Agenioideus saxigenus*** (Cockerell, 1908) **comb. nov.** (Figs 2A, 2B, 3A, 3B and 4A)

1908 *Agenia saxigena* Cockerell [9]: 229–230, fig 3.

1912 *Dipogon (Deuteragenia) saxigenus* (Cockerell); Sustera [10]: 191.

Type material. Compression fossil. Lectotype: USA, Colorado, Florissant Fossil Beds (UCMC No. 4541A) (Figs 2A and 3A). Paralectotype: USA, Colorado, Florissant Fossil Beds (UCMC No. 4541B) (Figs 2B and 3B). Examined.

Type stratum. Colorado Florissant Fossil Beds (Priabonian). The formation is a heterolithic accumulation of shale, tuffaceous mudstone and siltstone, tuff, and arkosic, volcaniclastic sandstone and conglomerate [11]. It is dated from the Priabonian, Eocene. Epis & Chapin [12] dated the formation from 34.9 Ma. Later, Evanoff *et al*. [11] analyses yielded a range of ages from 34.3 to 33.5 Ma. The most recent study [13] dated it from 33.7 to 34.7 Ma.

Diagnosis. Forewing hyaline, with two bands that cover 1Rs and 2Rs cells; maximum width 0.31X its length; 1Rs cell almost as long as 2Rs; 2m-cu vein slightly curved, meeting 2Rs cell 0.95X distance from base to apex of cell; and 2M cell with an inflection at the base of Cu.

Remarks. Two specimens collected from the same locality are found in the type series of this species. Some species of *Agenia* are currently classified under *Deuteragenia* Šustera, 1912. *Agenia saxigena* was placed in *Deuteragenia*, probably due to the double dark bands on the wings, which entirely cover the 1Rs and 2Rs cells (Figs 2A, 2B, 3A and 3B). Nevertheless, the inflection in the vein Cu at the base of the 2M cell identifies this specimen as Pompilinae. We place this species in *Agenioideus* because the 2m-cu vein of the forewing arises on the Cu vein much more than half the distance from the origin of 2M to the outer wing margin (Figs 2A and 3A). Other genera share this character (i.e. *Priocnemis* Schiodte, 1837; *Balboana* Banks, 1944; *Aplochares* Banks, 1944; and *Tachypompilus* Ashmead, 1902). This specimen differs from *Priocnemis* and *Balboana* by the presence of an inflection at the base of the Cu vein in 2M cell and by having long pronotum, which is short and angulate in the latter; and from *Tachypompilus* by the absence of irregular contours on the propodeum. Finally, this specimen has a large stigma, whereas in *Aplochares* the stigma is reduced. Because of its preserved morphological characters, and consequently, its taxonomic accuracy, this fossil can be used as a calibration point for stem-group *Agenioideus* (Fig 1, clade formed by *Agenioideus*, *Tachypompilus*, *Euryzonotulus*, *Homonotus* and *Ferreola*). This calibration point was used for divergence time estimation by Waichert *et al*. [2]. The fossil age falls at the upper bound of the estimated date error bar.

Genus ***Anoplius*** Dufour, 1834

***Anoplius planeta*** Rodriguez & Pitts, 2016

2016 *Anoplius planeta* Rodriguez & Pitts [1]: 2–4, fig 1.

Type material. Amber fossil. Holotype, OSAC Hy–10–45, 17–15 Ma, Dominican amber, DOMINICAN REPUBLIC, Cordillera Septentrional, between Puerto Plata and Santiago. Examined.

Type stratum. Dominican Amber (Burdigalian to Langhian). The age of Dominican Amber was established by Iturralde-Vinent [14] at “around” 16 Ma [1], based on the Midmiocene Climatic Optimum. This event has a possible age range from 17–15 Ma [15], and therefore we treat this as the age uncertainty for Dominican Amber.

Diagnosis. Antennae elongate, third antennal segment 1.8X as long as second segment; wings hyaline; maximum width 0.18X its length; 2Rs cell as long as 1Rs; 2m-cu vein slightly curved, meeting 2Rs cell 0.70X distance from base to apex of cell; and 2M cell with an inflection at the base of the vein Cu; base of metasoma paler, apical portion darker.

Remarks. This is the only *Anoplius* from Dominican amber. Rodriguez *et al*. (2016) confidently placed this species in *Anoplius* based on the following characters: postnotum as a transverse band with parallel anterior and posterior margins (Rodriguez *et al*. [1], fig 1b), 2m-cu vein arising on the Cu less than half the distance from the base of the 2M cell to the outer wing margin (Rodriguez *et al*. [1], fig 1c), clypeus emarginated, and claws bifid (Rodriguez *et al*. [1], fig 1d). Rodriguez *et al*. [1] discussed the difficulty of placing this species in any of the extant subgenera within *Anoplius*. Because this fossil can be confidently assigned to an extant genus, it can be used as a calibration point for the stem-group *Anoplius* (Fig 1, *Anoplius+Dicranoplius*).

Genus ***Tainopompilus*** Rodriguez & Pitts, 2016

***Tainopompilus argentum*** Rodriguez & Pitts, 2016

2016 *Tainopompilus argentum* Rodriguez & Pitts [1]: 5, fig 2.

Type material. Amber fossil. Holotype, OSAC Hy–10–45, 17–15 Ma, Dominican amber, DOMINICAN REPUBLIC, Cordillera Septentrional, between Puerto Plata and Santiago. Examined.

Type stratum. Dominican amber (Burdigalian to Langhian).

Diagnosis. Antennae slightly crenulated; wing hyaline; maximum width 0.31X its length; 2Rs cell as long as 1Rs; 2m-cu vein curved, meeting 2Rs cell 0.55X distance from its base to apex of cell; 2M cell with an inflection at the base of Cu vein; propodeal disc flat in profile view, posterior surface sloping abruptly at an angle.

Remarks. This is the only described species of *Tainopompilus*. It was placed in the subfamily Pompilinae by Rodriguez *et al*. [1] based on the presence of an inflection at the base of the vein Cu on 2M cell (Rodriguez *et al*. [1], fig 2b). It is morphologically similar to *Priochilus*, but the spine-like setae of uniform length on the metatibia and the inflection at the base of the vein Cu on 2M cell separate the two genera. Morphological convergence has been observed in several distantly related groups of Pompilidae that hunt in similar niches [2,16]. This may result from the ecological specificity in host use, which is phylogenetically conserved [17]. For these reasons, we abstain from assigning *T*. *argentum* to any stem group.

Genus ***Tenthredinites*** Meunier, 1915

***Tenthredinites bifasciata*** Meunier, 1915

1915 *Tenthredinites bifasciata* Meunier [18]: 11, fig 10.

Type material. Compression fossil. Holotype not available.

Type stratum. Aix-en-Provence fossil deposits (Chattian). The Aix-en-Provence basin is filled mainly with detritic sediments as well as limestone and gypsum [19]. Stratigraphic and floristic analyses of these deposits place it in the latest Oligocene (Chattian) 28.1–23 Ma [20,21].

Diagnosis. Forewing with two dark bands, maximum width 0.30X its length. The wing venation characters are not visible.

Remarks. The holotype of this specimen could not be located. The original description of this species does not mention its location and none of the museums contacted claimed to have it in their collection. Our only source of information is the species description and a blurry photograph included in the original publication. Meunier [18] mentioned the presence of this specimen in the Natural History Museum of Marseille. However, the curator did not respond to our inquiries concerning this specimen. Theobald [22] suggested a resemblance of this species to the extant *Pompilus maculipes* Smith, 1870, which is now placed in *Anoplius* [23]. We have no morphological evidence to place this species in *Anoplius*, and therefore we keep its original name.

### Pompilinae *incertae sedis*

***Pompilinites*** Rodriguez & Waichert **gen. nov.**

urn:lsid:zoobank.org:act:F5B5ABEC-55DC-4279-B46E-051E6BFAE746

Diagnosis. Wings hyaline with or without transverse dark spots; 2M cell with an inflection at the base of the Cu vein; legs with long spine-like setae set on grooves, spines uneven in length; metasoma without a transverse groove in the second sternite.

Description. Body length variable; forewing length variable. Head. Head wide, TFD > FD (Figs 2C and 3C); ocelli and mandible usually inconspicuous in fossils; flagellum elongate. Mesosoma. Pronotum elongated or not (Figs 2C and 3C); notauli present; propodeum variable; wing elongated; 2M cell with downward inflection at base of Cu vein; mid and fore femur with long setae in the apical margin; mid and fore tibia with long, uneven spine-like setae, setae set on grooves. Metasoma. Metasoma usually large, total length > total width.

Etymology. From Pompilin[ae] the latin suffix–ites “nature of, quality of”. This suffix is traditionally used for the generic epithet in fossils. The gender is masculine.

Remarks. This name is established as a collective-group name for all Pompilinae fossil species for which the generic position is unclear because of lack of diagnostic characters in preserved specimens. Pompilinae are promptly classified by the presence of long spine-like setae on metatibia and the Cu1 vein deflected downward at the base, with exceptions among species of *Priochilus* and *Balboana*. As a collective group, no description or diagnosis is provided. A type species is not required for collective groups ([24], art. 13.3.2, 42.3.1, 66, 67.14). Because of the uncertainty in their lower-level classification, all *Pompilinites* fossils can only be assigned to stem-group Pompilinae (Fig 1, divergence between Pompilinae and Pepsinae).

***Pompilinites coquandi*** (Theobald, 1937) **comb. nov.** (Figs 2C, 3C and 4B)

1937 *Pompilus coquandi* Theobald [22]: 320, pl. 24, fig 13; pl. 25, fig 18.

Type material. Compression fossil. Holotype: FRANCE, Bouches-du-Rhône, Aix-en-Provence (MNHN). Examined.

Type stratum. Aix-en-Provence deposits (Oligocene).

Diagnosis. Antennae long, third antennal segment 2.40X as long as wide; wing hyaline, maximum width 0.37X its length; 2Rs cell as long as 1Rs; 2m-cu vein straight, meeting 2Rs cell 0.50X distance from base to apex of cell; and 2M cell with an inflection at the base of the Cu vein.

Remarks. This species is unquestionably Pompilinae due to the presence of an inflection at the base of the Cu vein on the 2M cell (Fig 4B). The low quality of specimen preservation hinders an accurate identification to genus level.

***Pompilinites depressus*** (Statz, 1936) **comb. nov.**

1936 *Psammochares depressa* Statz [25]: 283, pl. 12, fig 34.

1945 *Pompilus depressus* (Statz); ICZN [24], opinion 166.

Type material. Compression fossil. Holotype not available.

Type stratum. Rott fossil deposits (Chattian), from Statz collection (northern Siebengebirge). The matrix of this deposit consists mainly of fine–grained paper shales, and thus contains very well-preserved fossils. Its age is controversial, ranging from the Chattian, Oligocene to the Aquitanian, Miocene [26]. More recent publications establish the Chattian as the correct age for this deposit [27].

Diagnosis. Wing hyaline; maximum width 0.31X its length; 1Rs cell 1.10X as long as 2Rs; 2m-cu slightly curved, meeting 2Rs cell 0.40X distance from base to apex of cell; and Cu vein of 2M cell with an inflection at its base.

Remarks. The generic position of this species is equivocal. We have only examined the wing venation illustrations in Statz [25] (pl. 12, fig 34), and the characters observed are dubious for determining its taxonomic status. Wing venation characters are not enough to determine the genus of this specimen. An inflection at the base of the Cu vein in the 2M cell is present, which places the species in the subfamily Pompilinae. Further inference would lead to an inaccurate taxonomic placement. The original description of this species does not indicate the location of the holotype and none of the natural history museums contacted claimed to have it in their collection.

Subfamily **Pepsinae** Lepeletier, 1845

Genus ***Chubutolithes*** Ihering (1922)

***Chubutolithes gaimanensis*** Bown & Ratcliffe, 1988

1988 *Chubutolithes gaimanensis* Bown & Ratcliffe [28]: 164–164, figs 3,4.

Type material. Ichnofossil. Holotype: ARGENTINA, Pan de Azucar, 1km west of Gaiman, Chubut Province, Sarmiento formation (MACH CH No. 1010). Not examined.

Type stratum. Sarmiento Formation (Priabonian). This formation is composed of alluvial rocks.

Remarks. *Chubutolithes* was described based on an ichnofossil composed of a mud nest. The structure of the pellets observed in the fossil suggests its affinity to *Auplopus* and its placement in Pompilidae [29–31]. Further analysis of this fossil suggests its formation on plant material and subsequent alluvial transport [29]. Simpson [32] studied the age of the entire Gaiman formation and concluded that the stratum where the holotype was found is Casamayoran (early Eocene) and not Patagonian (Miocene) as previously proposed by Schiller [33]. The stratigraphic distribution of the species is wide, ranging from Colhuehaupian (~20 Ma, [34]) to Casamayoran (55.8–48 Ma, [32]). According to Genise & Cladera [29] the only reliable fossils are from the same stratigraphical level as the holotype. The only other specimen from a younger stratum within the Sarmiento Formation (Colhuehaupian) shows signs of abrasion, which has been proposed as evidence of transport from older strata (i.e. Sarmiento Formation, [29]). If the age of *Chubulolithes* falls in the early Eocene, it would constitute the oldest Pompilidae fossil to date, which would have repercussions for the divergence-date estimates for the family, because the age is outside of the confidence interval obtained for Ageniellini (Fig 1). Nonetheless, recent analyses of fossil mammals from the Casamayoran revealed a much younger age in the late Eocene, Priabonian (36.6–35.3 Ma, [35]). The oldest sediments from the Sarmiento formation have been found to be no older than middle Eocene [36], rather than early Eocene as proposed previously. Therefore, the uncertainty in the age of Casamayoran fossils would impose a younger age range (36.6–35.3 Ma) on this calibration. Moreover, the fact that this is an ichnofossil adds taxonomic uncertainty, and even Evans & Shimizu [31]–to whom the confirmation of the assignment to Pompilidae has been attributed–have regarded it only as a “probable” *Auplopus-*looking nest. This uncertainty leaves open the possibility that this fossil derives from a much older ancestor within or even outside Pompilidae. Therefore, we do not recommend using this fossil in divergence-time analyses.

Genus ***Deuteragenia*** Šustera, 1912

***Deuteragenia catalunyia*** Rodriguez, Waichert & Pitts **sp. nov.** (Figs 2D and 3D)

urn:lsid:zoobank.org:act:073E375A-A1B7-4E15-951B-1545B0976D66

2001 *Dipogon (Deuteragenia)* sp. Arillo [37]: 80–82, fig 5.

Type material. Compression fossil. Holotype: SPAIN, Lleyda, Bellver de Cerdanya, deposit of Barranco de Salanca (MGMM No. 2927M). Examined.

Type stratum. Lacustrine deposits of Bellver, Spain (Messinian; [37]).

Diagnosis. Wing hyaline with two bands covering cells 1Rs, 2Rs, 2M and most of 2R1; maximum width 0.31X its length; 2Rs cell as long as 1Rs; 2m-cu vein almost straight, meeting 2Rs cell 0.40X distance from base to apex of cell; and 2M cell without an inflection at the base of Cu vein.

Description. Body length 9.10 mm; fore wing 7.50 mm; maximum wing width ~3.1 mm. Coloration. Integument black; wings hyaline black with two dark transverse bands; stigma black. Head. Head wide; TFD 0.93X FD (head slightly leaned); ocelli not preserved (Fig 3D). Mesosoma. Pronotum not well-preserved (Fig 2D); notauli present on 1/5 of anterior margin. Propodeum with lateral margins straight; propodeal disc; wing elongate; maximum width 0.31X its length; forewing hyaline with two bands covering cells 1Rs, 2Rs, 2M and most of 2R1; 2Rs cell as long as 1Rs; 2m-cu vein almost straight, meeting 2Rs cell 0.40X distance from base to apex of cell (Fig 2D). Metasoma. Metasoma 1.78X as long as wide; 1.70 X as long as mesosoma.

Etymology. This species is named after the autonomous community of Catalonia (Catalunyia in Catalan), where the fossil was found. The epithet is placed as a noun in apposition (ICZN [24], art. 31.2.1).

Remarks. This species was first studied by Arillo [37], who described it, but did not name it. We compared the holotype with the extant and extinct species of *Dipogon*, and name the new species herein. Because this species was found in Miocene deposits (younger than 11 Myr), the specimen was compared to all extant *Dipogon* (*Deuteragenia*) species [38,39] to confirm that it is a new species. Characters from wing venation were compared against all the other species. The most conspicuous difference found was the width of the 2R1 cell. *Dipogon catalunyia* sp. nov. has a 2R1 cell that is 4X as long as wide (Figs 2D and 3D), while in all other species it is less than 3X as long as wide (Arillo [37], fig 2). There are also differences in the distance from the beginning of the 1Rs where the 1m-cu vein is received at the base of 1Rs. Most of the described species do not receive this vein at 0.4X from the beginning of the 1Rs. Also, the distance where the 2m-cu vein is received by 2Rs is not 0.3X its length from the beginning of the cell in most species, as it is in *D*. *catalunyia* (e.g., 0.2X in *D*. *vechti* Day, 1979). Moreover, the extent to which the 2R1 cell is covered by dark banding, which in *D*. *catalunyia* is covered by 0.9X its length, is less in most of other species (e.g. 0.3X its length in *D*. *monticolus* Wahis, 1972, 1.0X its length in *D*. *subintermedius* [Maggretti, 1886]). Finally, the 1cu-a vein of the forewing meets the M+Cu slightly beyond the origin of the M, while in some species it meets M+Cu well beyond the origin of M (e. g., *D*. *austriacus* Wolf, 1964). This species can be distinguished from *Priocnemis* species, because in *Dipogon* the dark region covers the 1Rs and 2Rs cells completely, whereas in *Priocnemis* these are covered only partially [37]. Because of confidence in its taxonomic placement, this fossil species can be used as calibration point for *Deuteragenia* stem-group.

***Deuteragenia wettweri*** (Statz, 1938) **comb. nov. (**Figs 2E, 3E and 4C)

1938 *Priocnemis wettweri* Statz [40]: 108–109.

Type material. Compression fossil. Holotype: GERMANY, Rhineland-Palatinate, Rott (LACMIP No. 3973, LACMIP locality number 2533). Examined.

Type stratum. Rott deposits, Statz collection (northern Siebengebirge), dated from the Chattian [26].

Diagnosis. Wing with two bands, apex of hind wing darkened; maximum width 0.39X its length; 2Rs 1.2X longer than 1Rs; 2m-cu vein straight, meeting 2Rs cell 0.45X distance from base to apex of cell; and 2M cell without an inflection at the base of the Cu vein; metasoma 1.4X as long as mesosoma.

Remarks. The wing venation (Fig 4C) of this specimen resembles that of *Deuteragenia* species rather than *Priocnemis*. The 3r-m vein is curved in *Deuteragenia* (Fig 4C), whereas in *Priocnemis* it is straight; and the maximum width of 2Rs cell is greater than 2X its maximum length. Therefore, we transfer this species to *Deuteragenia*.

Genus ***Paleogenia*** Waichert & Pitts, 2016

***Paleogenia wahisi*** Waichert & Pitts, 2016

2016 *Paleogenia wahisi* Waichert & Pitts [1]: 3–4, fig 3.

Type material. Amber fossil. Holotype. RUSSIA, Kaliningrad Region, Baltic Sea (OSAC Hy–10–80). Examined.

Type stratum. Baltic amber. Fossils from the Kalinigrad region probably belong to the Prussian Formation, for which Kaplan *et al*. [41] suggested an absolute glauconite age of 37.7±3 Ma (Priabonian).

Diagnosis. Antennae short, third antennal segment 1.33X as long as second segment; apical antennal segment somewhat laterally compressed; wing hyaline (Rodriguez *et al*. [1], fig 2a1); maximum width 0.45X its length; cells short and rounded; 2Rs cell about the same size as 1Rs; 2m-cu vein slightly curved, meeting 2Rs cell 0.5X distance from base to apex of cell; 2R1 ending on apex of the forewing instead of anterior margin; and 2M cell without an inflection at the base of the Cu vein.

Remarks. Rodriguez *et al*. 2016 suggested that this species was probably a cleptoparasitoid, because of the short antennal segments with thick conspicuous setae (Rodriguez *et al*. [1], fig 2b1).

Genus ***Priocnemis*** Schiodte, 1837

***Priocnemis aertsi*** Statz, 1936 (Figs 2F, 3F and 4D)

1936 *Priocnemis aertsi* Statz [25]: 283–284.

Type material. Compression fossil. Holotype: GERMANY, Rhineland-Palatinate, Rott (LACMIP No. 3972, LACMIP locality number 2533). Examined.

Type stratum. Rott fossil deposits (Chattian), Statz collection (northern Siebengebirge).

Diagnosis. Wing hyaline with two dark bands, apex of wing darkened; maximum width 0.27X its length; 2Rs cell 1.20X longer than 1Rs; 2m-cu vein rounded, meeting 2Rs cell 0.33X distance from base to apex of cell; and 2M cell without an inflection at the base of the Cu vein.

Remarks. The original description of this species contained drawings of the wing venation and the entire specimen (Statz [25], pl. 12, figs 33–34). This species was placed in *Priocnemis* probably due to the presence of the 1cu-a vein extending beyond the M vein by about 0.70 to 1.30 of its length (Fig 4D). In other related Pepsini genera, this vein is closer to the M vein. Nevertheless, other Pepsini genera, such as *Entypus* Dahlbom, 1843, *Pepsis*, and *Calopompilus* Ashmead, 1900, also possess this character. The wing venation in *Priocnemis*, however, is distinguished from *Pepsis* by having the 2R1 cell ending straight, not separated apically from the costal margin of the wing, and from *Calopompilus* and *Entypus* by having the 2r-m vein slightly curved. In addition, it can be separated from *Dipogon* by the straight 3r-m vein, which is curved in *Dipogon*. Schoberlin [42] mentioned the presence of *Priocnemis* in the Oeningen deposits. However, the author did not provide a species description, or the location of the specimen mentioned. This fossil can be used as a calibration point for the stem-group *Priocnemis* (Fig 1).

Genus ***Caputelus*** Waichert & Pitts **gen. nov.**

urn:lsid:zoobank.org:act:53C64710-5D0D-403C-92F1-F1A65DB903C9

**Fig 5:**
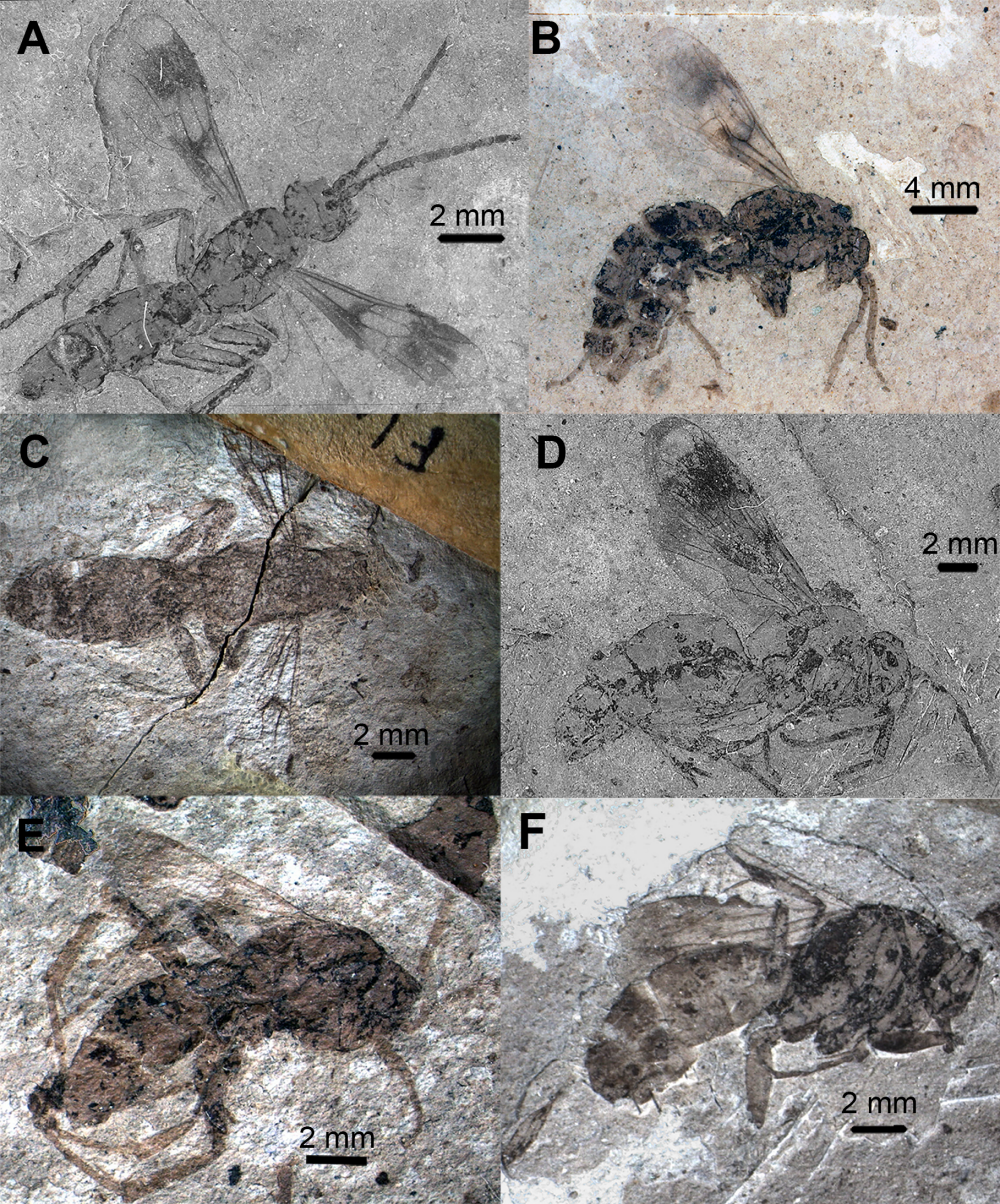
Photographs of *Caputelus scudderi*, *Cryptocheilus hypogaeus*, *Pepsinites avitula*, *Pepsinites florissantensis*, *Pepsinites laminarum* and *Pepsinites cockerellae* type specimens. Pompilidae fossils. A, dorsal view of *Caputelus scudderi* (Cockerell, 1906) from Florissant Fossil Beds, Colorado, US (MCZC No. 2024); B, habitus *of Cryptocheilus hypogaeus* Cockerell, 1914 from Florissant Fossil Beds, Colorado, US (USNM No. 90385); C, dorsal view of *Pepsinites avitula* (Cockerell, 1941) from Florissant Fossil Beds, Colorado, US (UCMC No. 19166); D, habitus of *Pepsinites florissantensis* (Cockerell, 1906) from Florissant Fossil Beds, Colorado, US (MCZC No. 2023); E, habitus of *Pepsinites laminarum* (Rohwer, 1909) from Florissant Fossil Beds, Colorado, US (UCMC No. 8597); F, habitus of *Pepsinites cockerellae* (Rohwer, 1909) from Florissant Fossil Beds, Colorado, US (UCMC No. 8598).

**Fig 6:**
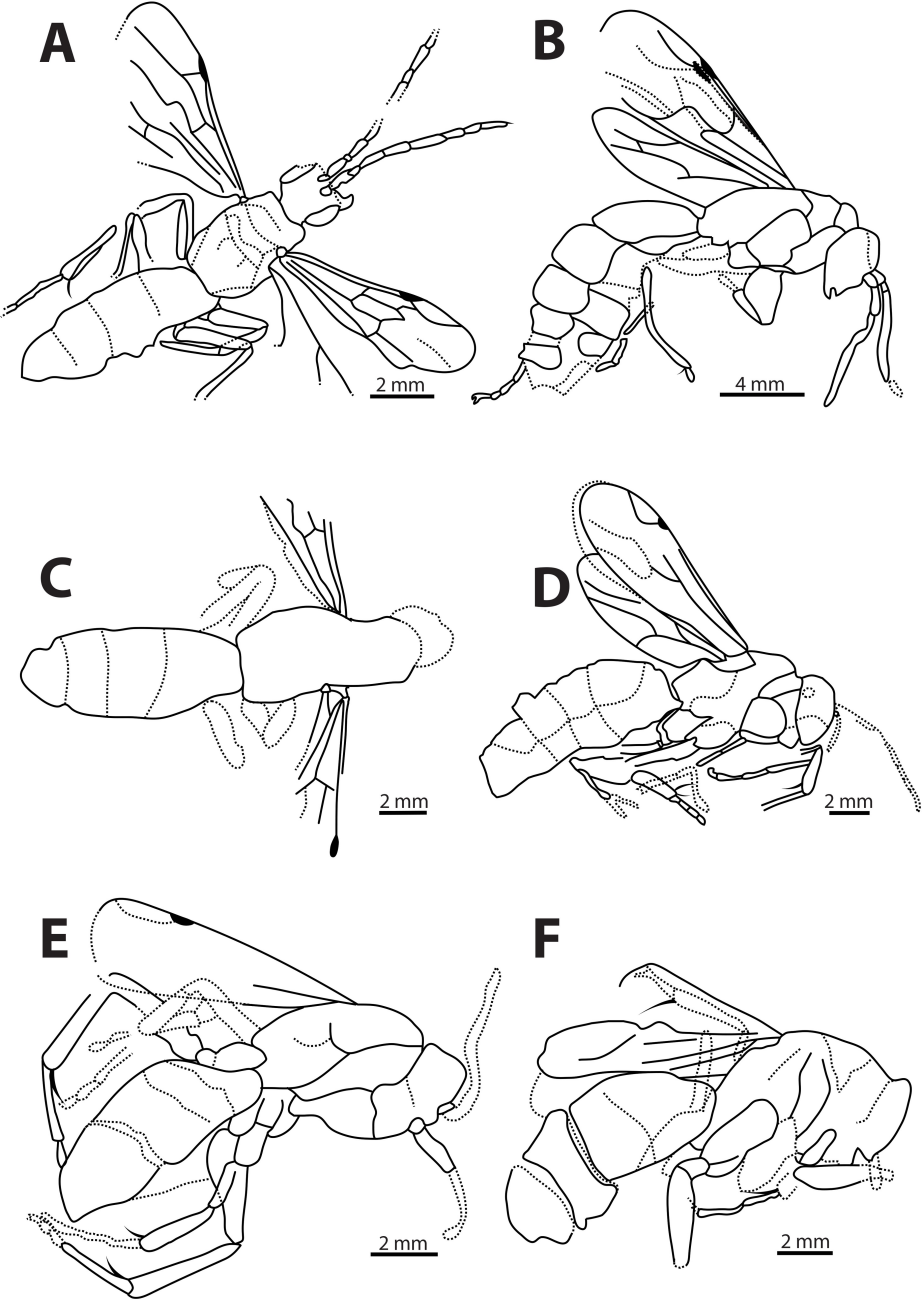
Line drawings of *Caputelus scudderi*, *Cryptocheilus hypogaeus*, *Pepsinites avitula*, *Pepsinites florissantensis*, *Pepsinites laminarum* and *Pepsinites cockerellae* type specimens. Pompilidae fossils. A, dorsal view of *Caputelus scudderi* (Cockerell, 1906) from Florissant Fossil Beds, Colorado, US (MCZC No. 2024); B, habitus *of Cryptocheilus hypogaeus* Cockerell, 1914 from Florissant Fossil Beds, Colorado, US (USNM No. 90385); C, dorsal view of *Pepsinites avitula* (Cockerell, 1941) from Florissant Fossil Beds, Colorado, US (UCMC No. 19166); D, habitus of *Pepsinites florissantensis* (Cockerell, 1906) from Florissant Fossil Beds, Colorado, US (MCZC No. 2023); E, habitus of *Pepsinites laminarum* (Rohwer, 1909) from Florissant Fossil Beds, Colorado, US (UCMC No. 8597); F, habitus of *Pepsinites cockerellae* (Rohwer, 1909) from Florissant Fossil Beds, Colorado, US (UCMC No. 8598).

***Caputelus scudderi*** (Cockerell, 1906) **comb. nov.** (Figs 5A and 6A)

1906 *Hemipogonius scudderi* Cockerell [43]: 53.

Type material. Compression fossil. Holotype: USA, Colorado, Florissant Fossil Beds (MCZC No. 2024). Examined.

Type stratum. Florissant Fossil Beds (Priabonian).

Type species. By monotype, *Caputelus scudderi* (Cockerell) comb. nov.

Diagnosis. Wing with two dark bands; maximum width 0.28X its length; 2Rs cell slightly longer than 1Rs; 2m-cu vein curved, meeting 2Rs slightly after its middle; and 2M cell without an inflection at the base of Cu vein; head wide, with two antennal pits; vertex extended posteriorly; pronotum short; leg spinose.

Description. Body length 12.00 mm. Forewing 7.00 mm; maximum wing width 2.00 mm. Head. Head wide, TFD 1.16X FH, vertex extended posteriorly (Figs 5A and 6A); ocelli in acute angle, lateral ocelli closer to each other than to compound eyes; first four antennal segments in the ratio 0.30: 0.34: 0.28: 0.24. Mesosoma. Mesosoma short, 0.75X as long as wide. Pronotum short, anterior margin straight, lateral edges rounded. Notauli present. Propodeum large, width 0.63X its length; propodeal posterior and lateral carina present. Forewing narrow (Figs 5A and 6A); maximum width 0.28X its length; length of 2R1 cell 0.60X the distance from its apex to wing margin; 2Rs cell 1.28X longer than 1Rs; two dark bands present. Leg without even spines distally; mid and hind tibia with scale-like spines; fore spur as long as antennal scape. Metasoma. Metasoma 2.94X as long as wide; 1.75X as long as mesosoma.

Etymology. The generic epithet comes from the Latin, *caput*-, which means head, and–*elus*, which refers to the suffix widely used for Pompilidae taxa. The name denotes the distinctive head shape of this taxon, which is extended in the vertex. The gender is masculine.

Remarks. *Caputelus* gen. nov. has the overall shape of the head, pronotum, and propodeum similar to those in extant species of Ctenocerinae, an Afrotropical subfamily. Additionally, the head has two dark spots, which could indicate it possessed antennal pits (Figs 5A and 6A), as diagnosed in Ctenocerinae. Extant genera of the New World, such as *Abernessia* Arlé, 1947 and *Lepidocnemis* Haupt, 1930, share similar morphological characteristics (see Waichert & Pitts with *Caputelus* gen. nov.[16,44]). *Abernessia* and *Lepidocnemis* were considered representatives of Ctenocerinae in the Neotropical Region until Waichert *et al*. [2] transferred these genera to Pepsinae based on molecular phylogenetic analysis. Waichert *et al*. [2] suggested that similarities between these Neotropical genera resulted from morphological convergence. The prolonged vertex and the antennal pit on the head, the large and carinate propodeum, and the absence of spines on legs suggest that *Caputelus* gen. nov. is morphologically related to *Abernessia* and *Lepidocnemis*, but differs from them by having the head with vertex well-prolonged; the pronotum short and angulate (it is long and rounded in the other genera); the 2R1 cell is large; and the forewing is banded.

*Caputelus* gen. nov. is here classified as Pepsinae, regardless of its similarities with Ctenocerinae, because it is more likely that these morphological features are the result of convergence, as it is for *Abernessia* and *Lepidocnemis*, than to consider that the Tertiary distribution of Ctenocerinae extended to the Northern Hemisphere.

***Cryptocheilus hypogaeus*** (Cockerell, 1914) (Figs 4E, 4F, 5B and 6B)

1914 *Cryptocheilus hypogaeus* Cockerell [45]: 718–719.

Type material. Compression fossil. Holotype: USA, Colorado, Miocene (sic) shales of Florissant, Wilson Ranch (H. F. Wickham) (USNM No. 90385). Examined.

Type stratum. Florissant Fossil Beds (Priabonian).

Diagnosis. Antennae long, third antennal segment 1.33X as long as second antennal segment; wings hyaline with two transverse dark spots; maximum width 0.33X its length; 2Rs cell longer than 1Rs; 2m-cu vein quite straight, meeting 2Rs slightly after middle; 1m-cu large and long, and 2M cell without an inflection at the base of the Cu vein; metasoma 1.6X as long as mesosoma.

Remarks. The wing venation is well preserved in this specimen. The absence of an inflection at the base of the Cu vein in 2M cell places it in the subfamily Pepsinae (Fig 4E). Furthermore, the robust body and the absence of a petiolate appearance in the first metasomal segment suggests that it belongs to the tribe Pepsini (Figs 5B and 6B). Finally, because the 2r-m vein is straight, and the cu-a ends distinctly before the juncture of M with Cu in the hindwing (Fig 4F), this species can be placed with confidence in the genus *Cryptocheilus*. Venation on forewing fades at the apex, probably because of poor preservation. This fossil can be used as a calibration point for stem-group *Cryptocheilus*.

***Pepsinites*** Rodriguez and Waichert **gen. nov.**

urn:lsid:zoobank.org:act:1D074E93-0DCE-4CE9-8ED3-716143EEE0CE

Diagnosis. Wings hyaline with or without two transverse dark spots; 2M cell without an inflection at the base of the Cu vein; legs with or without uniform spine-like setae; metasoma with a transverse groove in the second sternite.

Description. Body length variable; forewing varied. Head. Head wide, TFD > FD (Figs 5C–5F and 6C–6F); ocelli and mandible usually inconspicuous in fossils; flagellum elongate. Mesosoma. Pronotum not elongated (Figs 5C–5F and 6C–6F); notauli present; propodeum assorted. Wing elongate; 2M cell without inflection at base of Cu vein. Mid and hind tibia with or without short, sharpened spines (Figs 5C–5F and 6C–6F). Metasoma. Metasoma usually large, total length > total width; sternum 2 with transverse groove.

Etymology. From Pepsin[ae] the latin suffix–ites “nature of, quality of”. This suffix is traditionally used for the generic epithet in fossils. The gender is masculine.

Remarks. This name is established as a collective-group name for all Pepsinae fossil species for which the generic position is unclear due to lack of diagnostic characters in preserved specimens. Pepsinae are classified by having the mesofemur and the metafemur without subapical spine-like setae set in grooves or pits; the metatibia with uniform apical spine-like setae; and the fore wing with vein Cu1 without a posterior downward deflection (pocket). As a collective group, a type species is not required for collective groups ([24], art. 13.3.2, 42.3.1, 66, 67.14). Because of the uncertainty in their lower-level classification, all *Pepsinites* fossils can only be assigned to stem-group Pepsinae (Fig 1).

***Pepsinites scelerosus*** (Meunier, 1917) **comb. nov.**

1917 *Pompilus scelerosus* Meunier [18]: 181–184.

Type material. Amber fossil. Holotype not available.

Type stratum. Baltic amber (Lutetian to Priabonian). The exact locality is unknown. Baltic amber deposits have been obtained for more than 100 years, and their age is controversial. Microfaunistic dating of the deposits containing the largest amount of amber suggest they are from the Priabonian, Eocene (37.7 Ma) (Kaplan *et al*. [41]), whereas radiometrically dated glauconite as Lutetian, Eocene (47.0 to 44.1 Ma) [46]. Perkovsky *et al*. [47] considered the Ritzkowski [46] data insufficient to disprove Kaplan [41], because the former was based on two samples and the latter on seven samples. More recent data indicate that the age of Baltic Amber can be narrowed to 34 to 38 Ma based on palynological data [48,49].

Diagnosis. Wing hyaline, maximum width 0.26X its length; 2Rs cell 1.29X longer than 1Rs; 2m-cu vein straight, meeting 2Rs cell 0.40X distance from base to apex of cell; and 2M cell without an inflection at the base of Cu vein.

Remarks. This is a very small specimen from Baltic amber that does not have an inflection at the base of the Cu vein of the 2M cell, which excludes it from Pompilinae. The description and drawings provided by Meunier ([50], figs 1–3) suggest that this specimen should be placed in Pepsinae. The location of the holotype of this species was not mentioned in the original description and none of the museums contacted claimed to have it in their collection. Because we could not locate the holotype, and the published drawing is inadequate, we cannot make further taxonomic conclusions about this taxon.

***Pepsinites avitula*** (Cockerell, 1941) **comb. nov.** (Figs 4G, 5C and 6C)

1941 *Pepsis avitula* Cockerell [51]: 355–356, pl. 1, fig 3.

2005 *Chirodamus avitula* (Cockerell); Vardy [52], p. 285, fig 688.

Type material. Compression fossil. Holotype: USA, Colorado, Florissant Fossil Beds (UCMC No. 19166). Examined.

Type stratum. Florissant Fossil beds (Priabonian).

Diagnosis. Wing hyaline, banded; maximum width 0.35X its length. Other wing venation characters are not visible.

Remarks. Vardy [52] studied this specimen, and mentioned a number of characters that differentiate it from *Pepsis*. He also mentioned the lack of *Pepsis* species that possess banded wings, and suggested the proximity of this species to *Chirodamus* Haliday, 1837, thus establishing the combination. We did not find enough wing venation characters to justify its placement in *Chirodamus* (Fig 4G). Therefore, we place this species in the collective group *Pepsinites*.

***Pepsinites florissantensis*** (Cockerell, 1906) **comb. nov. (**Figs 5D and 6D)

1906 *Hemipogonius florissantensis* Cockerell [43]: 52–53.

1914 *Cryptocheilus florissantensis* (Cockerell); Cockerell [45]: 719.

Type material. Compression fossil. Holotype: USA, Colorado, Florissant Fossil Beds (MCZC No. 2023). Examined.

Type stratum. Florissant Fossil beds (Priabonian).

Diagnosis. Wing hyaline with two dark bands, apex darkened; maximum width 0.20X its length; 2Rs cell slightly shorter than 1Rs; 2m-cu vein slightly curved, meeting 2Rs cell 0.70X distance from base to apex of cell; 2M cell without an inflection at the base of Cu; and hindwing with cu-a ending distinctly before the juncture of M with CU.

Remarks. The specimen was preserved with wings overlapping (Figs 5D and 6D), which precluded accurate description and illustration of wing venation characters. Nevertheless, Cockerell [43] provided a good description and several measurements of hindwing cells. The placement of *C*. *florissantensis* in *Hemipogonius* Cockerell, 1906 was not justified by Cockerell [43]. The same author later placed the species within *Cryptocheilus* [45]. The generic position of this species cannot be certain because wing venation characters are not entirely visible. It certainly belongs to Pepsinae, because of the absence of an inflection at the base of the Cu vein in 2M cell.

***Pepsinites laminarum*** (Rohwer, 1909) **comb. nov.** (Figs 5E and 6E)

1909 *Salius laminarum* Rohwer [53]: 26–27.

1914 *Cryptocheilus laminarum* (Rohwer); Cockerell [45], p. 718.

Type material. Compression fossil. Holotype: USA: Colorado: Florissant, Fossil Beds, Station 14, 1908 (S. A. Rohwer) (UCMC No. 8597). Examined.

Type stratum. Florissant Fossil Beds (Priabonian).

Diagnosis. Wing hyaline with apex darkened; maximum width 0.26X its length; 2Rs cell almost as long as 1Rs; and 2m-cu and 2M cell not observable.

Remarks. Cockerell [45] placed this species in *Cryptocheilus*. The wing venation is not well preserved, but the robust body, flat metasoma, and the absence on an inflection at the base of the Cu vein of 2M cell likely places it in the tribe Pepsini (Figs 5E and 6E). Characters that might assign it to a particular genus within the tribe are not visible.

***Pepsinites contentus*** (Theobald, 1937) **comb. nov.**

1937 *Criptochilus contentus* (*sic*) Theobald [22]: 129–130.

Type material. Compression fossil. Holotype: FRANCE, Gard, Célas (MHNN). Not examined.

Type stratum. Terrains sannoisiens du Gard, France (Priabonian). The age of this deposit has been addressed in various recent publications that refer to the Theobald specimens [54,55], but the source of the age justification is not addressed and is, so far, unknown to us. The only age range has been mentioned as 37.2–33.9 Ma [54], falling within the Priabonian. Collomb et al. [55] set the date as “Late Priabonian, 35 Mya, Gard, France” and Perrard *et al*. [56] suggest a “Late Eocene” date based on the French Geological Survey (Bureau de Recherches Géologiques et Minières) geological map 1/50 000, number 912. There is no other mention of these deposits and their age in recent literature.

Diagnosis. Wing hyaline; maximum width 0.31X its length; 2Rs cell 1.5X longer than 1Rs; 2m-cu vein straight, meeting 2Rs cell 0.33X distance from base to apex of cell; and 2M cell without an inflection at the base of the Cu vein.

Remarks. This species was described as “*Criptochilus contentus*”, with a misspelling in the generic name, and was not mentioned by Engel & Grimaldi [57] in their revision. The venation of the hindwing was not illustrated by Theobald [22], but based on the forewing it belongs unquestionably to Pepsinae. However, the lack of information on the hindwing venation precludes its accurate generic placement.

***Pepsinites cockerellae*** (Rohwer, 1909) **comb. nov.** (Figs 5F and 6F)

1909 *Agenia cockerellae* Rohwer [53]: 24–25.

1912 *Dipogon (Deuteragenia) cockerellae* (Rohwer); Sustera [10]: 191.

2012 *Deuteragenia cockerellae* (Rohwer); Lelej [58]: 7–9.

Type material. Compression fossil. Holotype: USA, Colorado, Florissant Fossil Beds, Station No. 11 (North End of Stump Hill) (UCMC No. 8598). Examined.

Type stratum. Florissant Fossil Beds (Priabonian).

Diagnosis. Wing hyaline, with two dark bands; maximum width 0.16X its length; and 2M cell without an inflection at the base of the Cu vein.

Remarks. This species was placed in the subgenus *Deuteragenia* based on the wing-banding patterns (Figs 5F and 6F). Many pompilid genera in the two most species-rich subfamilies (e.g. *Priochilus* and *Ageniella* Banks, 1912), possess dark areas on the wings, which makes it an ambiguous character. We conclude that the current generic position of this species is equivocal, and the preservation of the fossil does not allow us to draw any further conclusions. The main wing venation characters of Pepsinae, however, are present. Therefore, we place this species in *Pepsinites*.

**Pompilidae** *Incertae sedis*

***Pompilite****s* Rodriguez **gen. nov.**

urn:lsid:zoobank.org:act:D1682AE0-9768-44D3-BBEC-FD22F6427F0C

Diagnosis. Wings hyaline with or without transverse dark spots; forewing with ten closed cells; the veins C, 2rs-m, 3rs-m, 2m-cu, 2-RS are present, 1cu-a closer to vein 1M than to its junction with Cu vein; hindwing with distinct claval lobe absent, first abscissa of Cu present, 1A vein absent, 1rs-m crossvein absent, second abscissa of M present, and the veins C+Sc+R+Rs fused basally.

**Fig 7:**
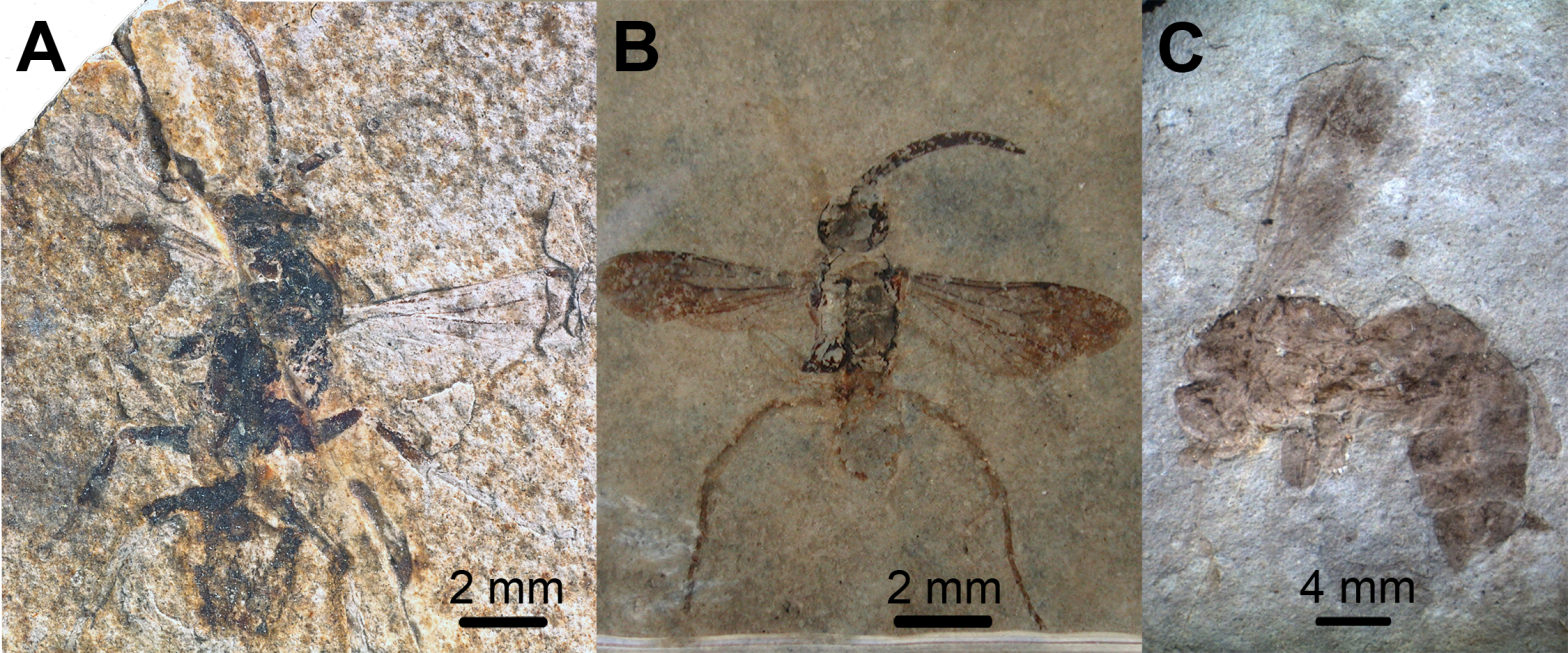
Photographs of *Pompilites induratus*, *Pompilites fasciatus* and *Pompilites senex* type specimens. A, habitus of *Pompilites induratus* (Heer, 1849) from Oeningen, Germany (SMNK); B, dorsal view of *Pompilites fasciatus* Theobald, 1937 from Aix-en-Provence, France (MNHN); C, habitus of *Pompilites senex* (Rohwer, 1909) from Florissant Fossil Beds, Colorado, US (UCMC. No. 8594).

**Fig 8:**
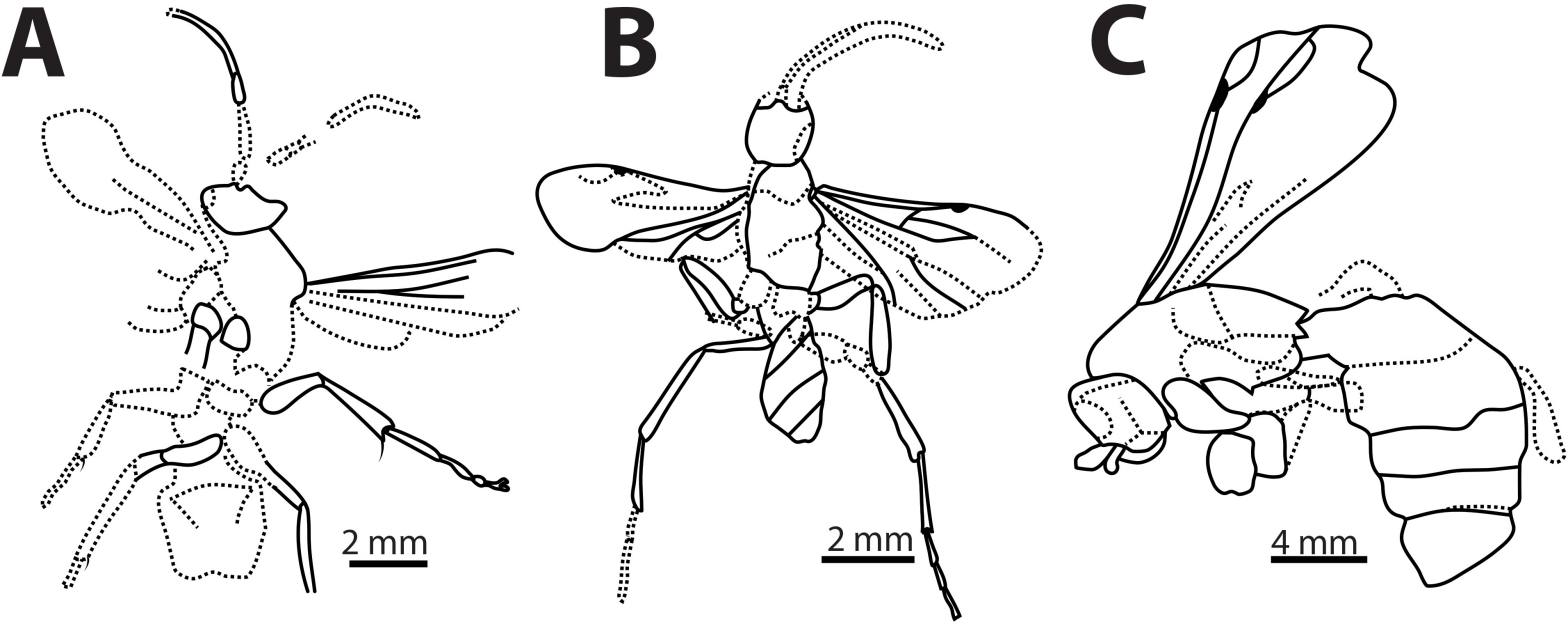
Line drawings of *Pompilites induratus*, *Pompilites fasciatus* and *Pompilites senex* type specimens. A, habitus of *Pompilites induratus* (Heer, 1849) from Oeningen, Germany (SMNK); B, dorsal view of *Pompilites fasciatus* Theobald, 1937 from Aix-en-Provence, France (MNHN); C, habitus of *Pompilites senex* (Rohwer, 1909) from Florissant Fossil Beds, Colorado, US (UCMC. No. 8594).

Description. Description. Body length varied; forewing varied. Head. Head wide; TFD > FD; punctuation inconspicuous; ocelli and mandible usually inconspicuous in fossils. Mesosoma. Pronotum usually not elongated (Figs 7A–7C and 8A–8C); notauli present; propodeum assorted; wing elongate; forewing with ten closed cells, C vein present, 1Rs not directly joining pterostigma, abscissa distad Rs + M from M vein present, veins 2rs-m and 3rs-m present, 2m-cu present, 1cu-a closer to vein 1M than to its junction with Cu vein, 2-Rs vein present; hindwing with distinct claval lobe absent, first abscissa of Cu present, 1A vein absent, 1rs-m crossvein absent, second abscissa of M present, and the veins C+Sc+R+Rs fused basally. Mid and fore tibia with or without spines set on grooves (Figs 7A–7C and 8A–8C). Metasoma. Metasoma usually elongate.

Etymology. From Pompil[idae] the latin suffix–ites “nature of, quality of”. This suffix is traditionally used for the generic epithet in fossils. The gender is masculine.

Remarks. This name is established as a collective-group name for all Pompilidae fossil species for which the subfamilial and generic position is unclear because of lack of diagnostic characters in preserved specimens. As a collective group, a type species is not required for collective groups ([24], art. 13.3.2, 42.3.1, 66, 67.14). Because of lack of evidence to place them in extant subfamilies, these are currently assigned to crown-group pompilidae, but not recommended for in phylogenetic time-calibrations (Fig 1).

***Pompilites induratus*** (Heer, 1849) **comb. nov.** (Figs 7A and 8A)

1849 *Pompilus induratus* Heer [59]: 165–166, fig 10, pl. 13.

1909 *Anoplius induratus* (Heer); Rohwer [53]: 28.

Type material. Compression fossil. Holotype: GERMANY, Baden-Württemberg, Wangen im Allgäu, Oeningen (SMNK). Examined.

Type stratum. Oeningen fossil beds (Langhian to Serravallian). This region had first been reported as belonging to Switzerland. Cockerell [43] corrected it and located the region in Baden-Württemberg, Germany. Oeningen is one of the richest insect fossil deposits known in the world. It is composed of freshwater limestone deposits that date from the Langhian to the Serravallian (15–11 Ma), based on stratigraphic data and terrestrial insect sediment analysis [60–62,27].

Diagnosis. Wing hyaline, maximum width 0.29X its length; 1Rs triangular, small; and 2M cell and 2m-cu vein not visible.

Remarks. We are doubtful about the subfamilial and generic classification of this species, because of the poor preservation of the specimen (Figs 7A and 8A). Rohwer [53] transferred this species to *Anoplius*, but made no comments on the reasons for this decision. Characters that would place it in the subfamily Pompilinae, such as the inflection on the base of Cu vein in the 2M cell or the spines of different lengths on the apex of the metatibia, cannot be observed, nor can diagnostic characters of other subfamilies.

***Pompilites fasciatus*** (Theobald, 1937) **comb. nov.** (Figs 4H, 7B and 8B)

1937 *Pompilus fasciatus* Theobald [22]: 320, pl. 24, fig 14; pl. 25, fig 14.

Type material. Compression fossil. Holotype: FRANCE, Bouches-du-Rhône, Aix-en-Provence (MNHN). Not examined.

Type stratum. Aix-en-Provence fossil deposits (Chattian).

Diagnosis. Antennae short, antennal segments wide; wing hyaline, darkened towards the apex in about 1/3 of the total length, maximum width 0.35X its length; 2Rs cell 1.20X longer than 1Rs; 2m-cu vein slightly curved, meeting 2Rs cell 0.60X distance from base to apex of cell; and 2M cell with an inflection at the base of Cu vein.

Remarks. The general morphology of the specimen is very similar to the description of extant cleptoparasitic Pompilidae by Shimizu [63]. Pompilidae cleptoparasites typically have shortened antennal segments (Figs 7B and 8B), such as in *Aridestus* Banks, 1947, or *Poecilagenia* Haupt, 1927 [63]. Nevertheless, cleptoparasites are known in three subfamilies, and the accurate generic placement of this specimen is not possible due to the lack of detail in preserved structures.

***Pompilites incertus*** (Theobald, 1937) **comb. nov.**

1937 *Pompilus incertus* Theobald: 284, pl. 20, fig 2; pl. 7, fig 4.

Type material. Compression fossil. Holotype: FRANCE, Camoins-les-Bains (MHNM). Not examined.

Type stratum. Camoins-les-Bains fossil deposits (Chattian). These deposits have been dated from the latest Oligocene (Chattian) by stratigraphic, sedimentary and paleogeographic reconstructions [64].

Diagnosis. Not applicable.

Remarks. The holotype of this specimen could not be studied because it was not possible to locate the museum curator. The images in the original description of the species do not allow classifying this specimen in any of the Pompilidae genera or subfamily, therefore we include it in *Pompilites*.

***Pompilites senex*** (Rohwer, 1909) **comb. nov.** (Figs 7C and 8C)

1909 *Salius senex* Rohwer [53]: 25–26.

1914 *Cryptocheilus senex* (Rohwer); Cockerell [45]: 718.

Type material. Compression fossil. Holotype: USA, Colorado, Florissant, Fossil Beds, Station 14, 1908, collector unknown (UCMC. No. 8594). Examined.

Type stratum. Florissant Fossil Beds (Priabonian).

Diagnosis. Wing hyaline with two dark bands; maximum width 0.30X its length; 2Rs longer than 1Rs; 2m-cu slightly curved, meeting 2Rs slightly before the middle; 2M cell not observable; and metasoma 1.51X mesosoma.

Remarks. Even though Rohwer [53] mentions the affinity of *C*. *senex* with *Anoplius* species, the poor wing preservation precludes a confident designation of this species in *Anoplius*. It is possible, however, that *C*. *senex* is a junior synonym of *C*. *florissantensis* (Cockerell) due to the overall body shape, wing coloration, and collection site. Nevertheless, given the poor preservation of the wing venation and absence of legs (Figs 7B and 8B), it is not possible even to assign a subfamily for *C*. *senex*.

Family **Aulacidae**

Genus ***Ceropalites*** Cockerell, 1906

***Ceropalites infelix*** Cockerell, 1906

1906 *Ceropalites infelix* Cockerell: 53–54.

Remarks. One of the previously described compression fossils from Florissant, Colorado, does not belong to Pompilidae. This is the case of *Ceropalites* Cockerell, 1906, which was described based on a single specimen, *C*. *infelix* Cockerell, 1906. Upon examining this Florissant fossil, we noticed that the holotype had a second label “Aulacidae, Rasnitsyn 1989”. To our knowledge, this new status has remained unpublished. We agree with Rasnitsyn’s placement of this species. Herein we transfer *C*. *infelix* to Aulacidae based on analyses of wing venation, petiole, and mesosoma, but refrain from making taxonomic decisions about its generic placement. The wing has a very large cell 2R1, which extends to the apex of the wing. This is uncommon in Pompilidae. Additionally, the cell 2Rs is short and 3Rs is not even vestigial in the fossil; cell 1M does not match the typical venation in Pompilidae, but rather the venation observed in Evanioidea. Furthermore, this species has a well-defined petiole and the metasoma seems to be raised from the metanotum, as observed in taxa of Aulacidae and absent in Pompilidae. In addition, the propodeum is short and the integument is rugose, unlike any extant species of spider wasps.

### Fossils and divergence time estimation

Accuracy in fossil identification and age has become increasingly relevant for molecular phylogenetic studies due to the utility of fossils as calibration points for dating phylogenies. Many times, fossil information is being used by systematists in the absence of appropriate taxonomic revisions of fossil taxa, thereby providing erroneous time estimates that can lead to wrong conclusions about diversification, biogeography, etc. Consequently, current revisions of fossil taxa for higher taxonomic groups become imperative.

Another issue arises because of the misuse of stratigraphic information, which may increase uncertainty in age estimates. This becomes more apparent in younger groups like Pompilidae, where the most accurate fossil identifications are from amber deposits, for which the age is controversial with wide time ranges.

Pompilidae is one of the least taxonomically studied Hymenoptera families. One of the main reasons for the paucity of systematics research is the difficulty in identification stemming from morphological uniformity even between subfamilies. Recent molecular and paleontological studies place the origin of the family in the Eocene [1,2], with origin of crown-group subfamilies no later than the Oligocene. The morphological homogeneity, together with limited amount of preserved structures, makes fossil Pompilidae identification highly inaccurate, especially for compression fossils.

Our study is the first attempt at clarifying the classification of fossil spider wasps. From the 17 fossil species described, only eight can be determined to genus with confidence. The remaining species have been placed in collective-group genera that indicate the uncertainty in taxonomic placement and avoid further confusion in fossil Pompilidae ages.
